# Supplementary material for: MicroDiffuse3D: A Foundation Model for 3D Microscopy Imaging Restoration
Source: ArXiv. 2026 May 8:arXiv:2605.08566v1. Preprint. [Version 1] (PMC13178444)
Supplement: 1 [file NIHPP2605.08566V1-supplement-1.pdf]

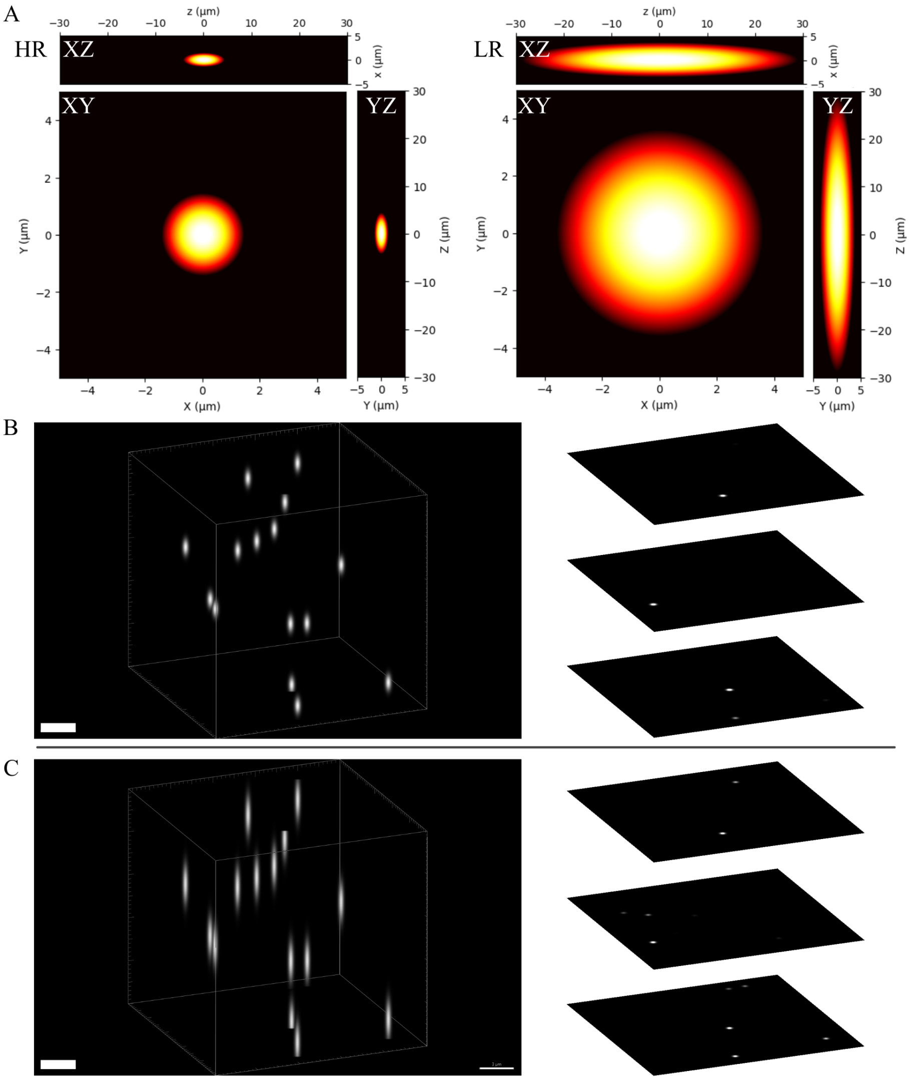

Supplementary Figure 1: **Point spread function illustration. A, Differences in the point spread function between high-resolution (HR) and low-resolution (LR) objectives.** The LR objective has a more spatially dispersed kernel, yielding a broader effective collection profile and greater axial signal entanglement. This causes each voxel to contain more mixed volumetric information and makes ideal slice-wise reference or alignment between LR and HR acquisitions difficult. **B, C, Simulated examples of this effect. B,** simulated 3D representation of point sources acquired with the HR objective, with cross-sections from the top, middle, and bottom shown on the right. **C,** simulated 3D representation of the same objects using LR acquisition parameters. Scale bar 5 microns.

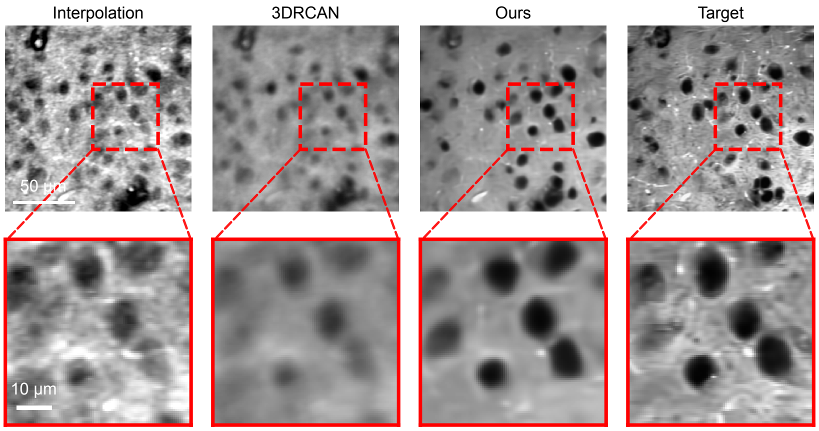

Supplementary Figure 2: **Visual comparison of super-resolution quality.** Representative images of the low-quality input, 3DRCAN, our method, and the high-quality target, with enlarged views shown below. Our reconstruction shows clearer nuclear appearance and better-defined biological structure boundaries.

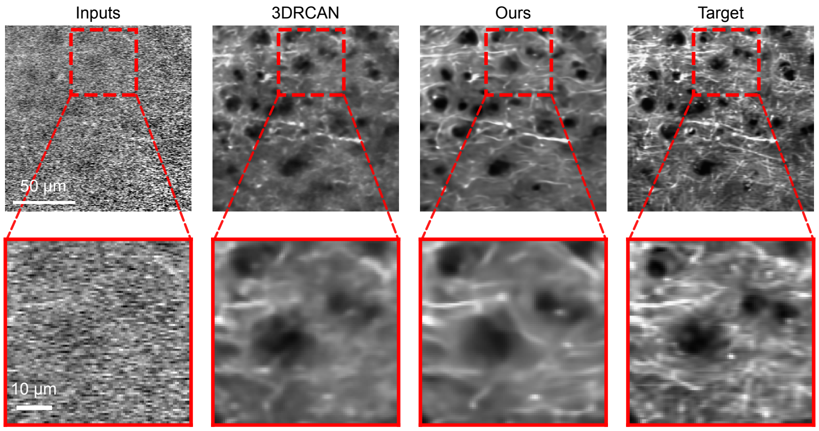

Supplementary Figure 3: **Visual comparison of denoising quality.** Representative images of the low-quality input, 3DRCAN, our method, and the high-quality target, with enlarged views shown below. Our reconstruction shows improved structural clarity and better-defined biological boundaries.

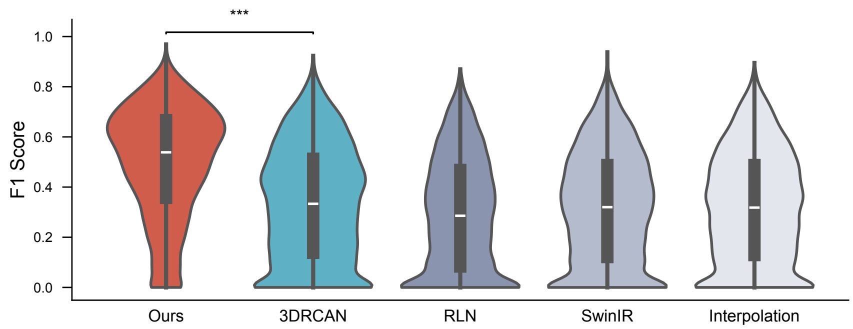

Supplementary Figure 4: **Cell segmentation performance measured by F1 score.** Quantitative comparison of segmentation quality across different reconstruction methods. Our method achieves the highest F1 score among all methods. Statistical significance was determined using the Wilcoxon signed-rank test.

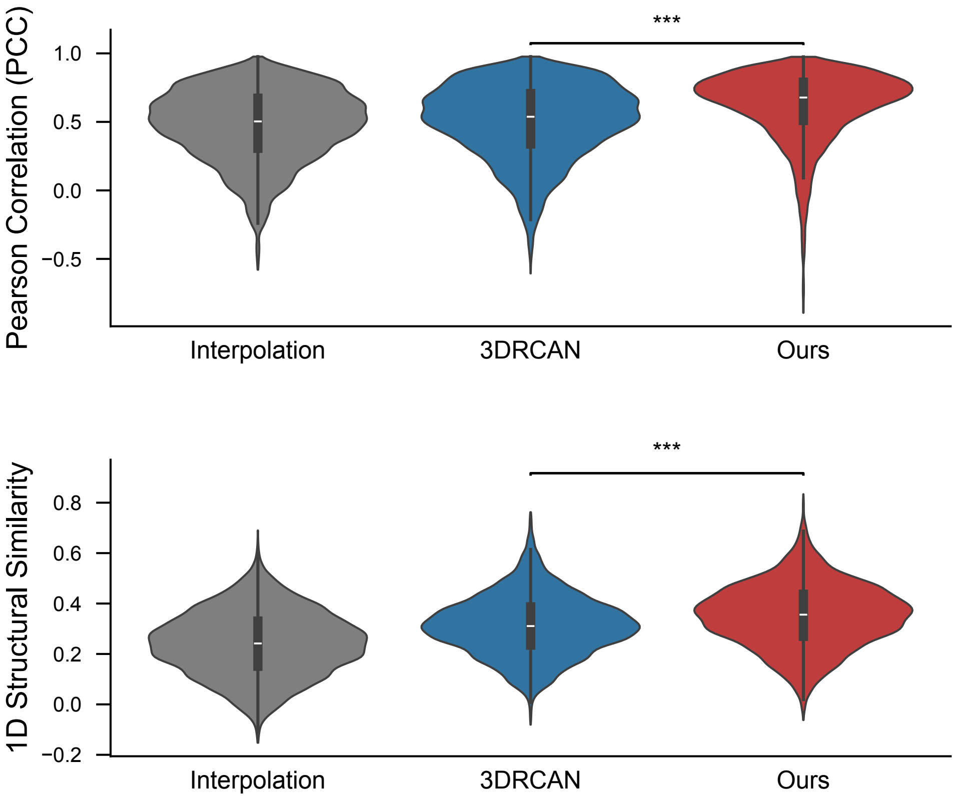

Supplementary Figure 5: **PCC and 1D SSIM of reconstructed signal profiles. a,** PCC between reconstructed and target intensity profiles. **b,** 1D SSIM between reconstructed and target intensity profiles. Our method shows the highest profile consistency with the target. Statistical significance was determined using the Wilcoxon signed-rank test.

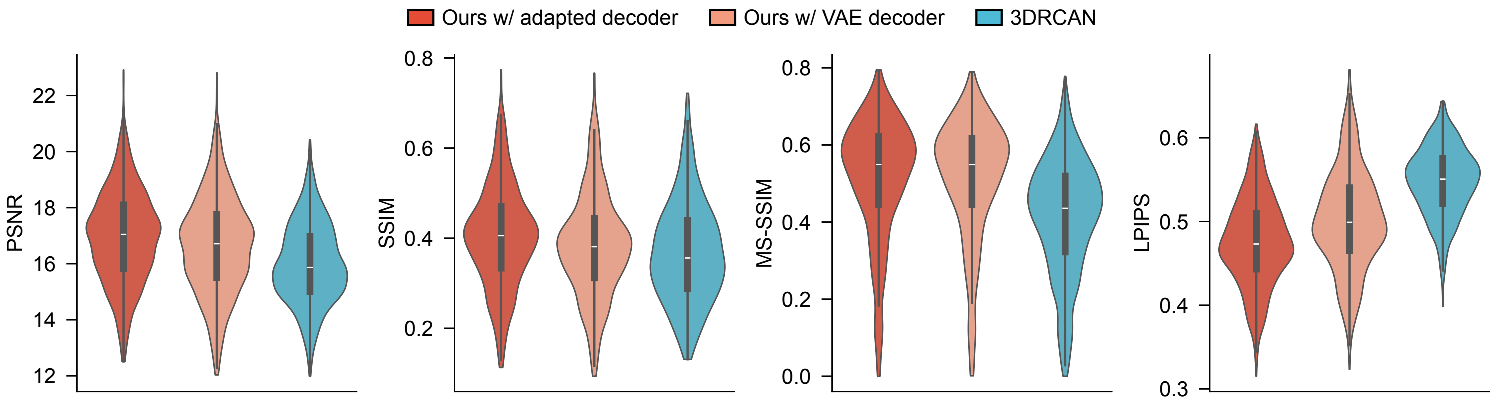

Supplementary Figure 6: **Performance comparison of different decoders and 3DRCAN.** Comparison of PSNR, SSIM, MS-SSIM, and LPIPS for MicroDiffuse3D with the adapted decoder, MicroDiffuse3D with the original VAE decoder, and the standard 3DRCAN restoration model. Notably, MicroDiffuse3D with the original VAE decoder already substantially outperforms 3DRCAN. The adapted decoder further improves reconstruction quality over the original VAE decoder.

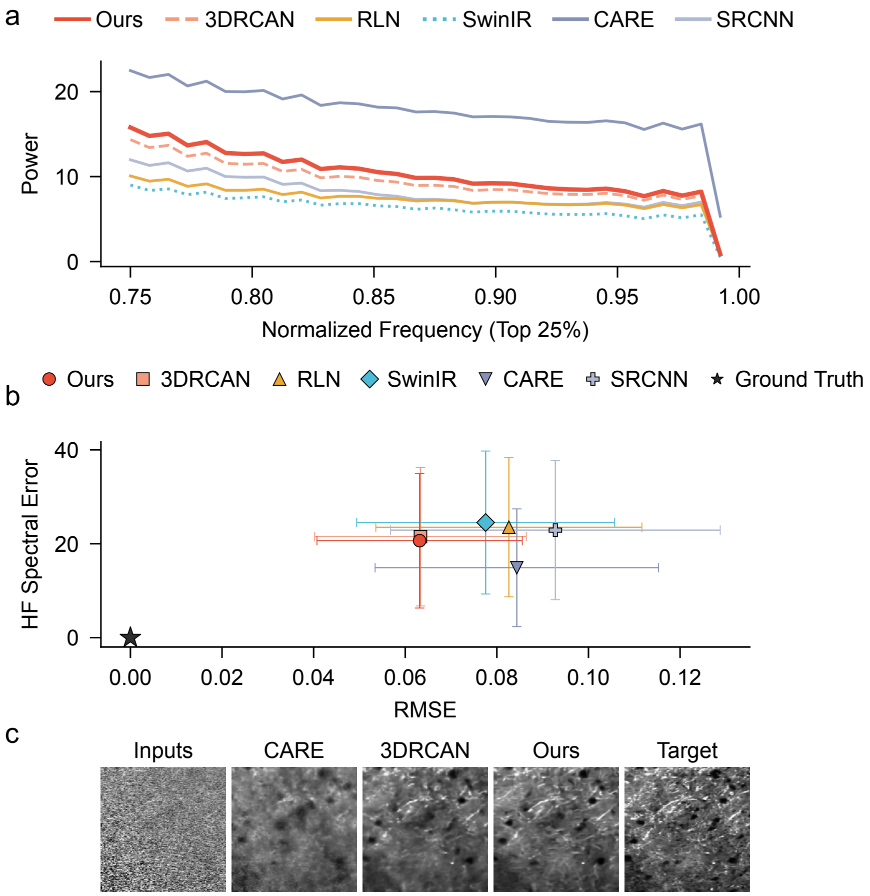

Supplementary Figure 7: **Spectral analysis and perception-distortion trade-off. a, High-frequency radial power spectrum.** Averaged power spectrum of the top 25% frequencies (normalized by Nyquist frequency). Our method resolves sharper anatomical features by retaining substantially higher spectral energy than most baselines. The ground truth is omitted to preserve the visualization scale due to its significantly higher energy. The higher energy observed in CARE reflects noise artifact, evidenced by its high voxel-wise error. **b, Perception-distortion evaluation.** Scatter plot of High-Frequency (HF) Spectral Error versus Root Mean Square Error (RMSE). HF Spectral Error is defined as the average of absolute power difference with the ground truth. Our approach achieves the best voxel-wise fidelity while maintaining the most competitive HF spectral error, positioning it closest to the ideal Ground Truth (black star). Error bars indicate standard deviation across the test volumes. **c, Visual comparison between CARE, 3DRCAN and MicroDiffuse3D.** CARE fails to fully remove gaussian noise, due to its residual connection and limited denoising capability in extreme scenario, which explain the abnormal high-frequency power in its generation. MicroDiffuse3D provides better fine-grained details in comparison with 3DRCAN.

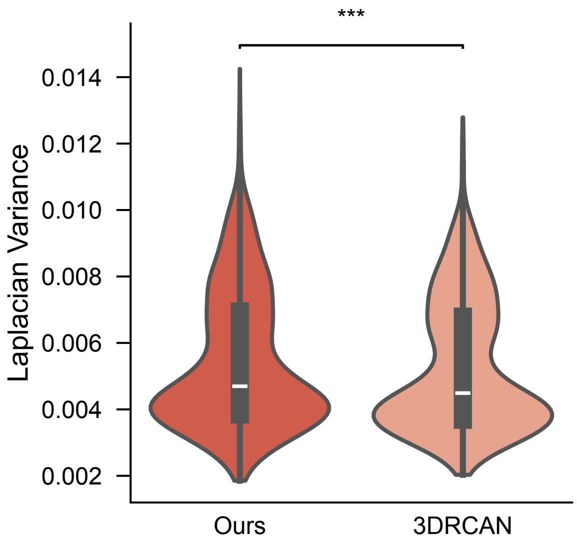

Supplementary Figure 8: **Comparison of Laplacian variance.** Our method resolves sharper anatomical features by retaining substantially higher Laplacian variance than the representative baseline. Statistical significance was determined using the Wilcoxon signed-rank test.
